# Individual variation within wild populations of an arid-zone lizard dictates oxidative stress levels despite exposure to sublethal pesticides

**DOI:** 10.1007/s10646-023-02653-8

**Published:** 2023-04-26

**Authors:** Isabella Contador-Kelsall, Kimberly Maute, Maxwell de Beer, Kristine French

**Affiliations:** 1 School of Earth, Atmospheric and Life Sciences, University of Wollongong, Northfields Ave, Wollongong, 2522 NSW Australia; 2 Department of Planning and Environment, Ecosystems and Threatened Species, Biodiversity and Conservation, PO Box 2011, Dubbo, NSW 2830 Australia; 3 Wollongong, 2500 NSW Australia

**Keywords:** Arid-zone, DNA damage, Lizard, Oxidative stress, Pesticide, Protein carbonyl

## Abstract

The relationship between sublethal pesticide exposure and oxidative stress in an ecologically relevant field setting is relatively unknown for reptiles. Oxidative stress is a multi-faceted concept that dictates key survival and fitness parameters in any organism. Fipronil and fenitrothion are two pesticides widely used globally for agricultural pest management. Using a field-based, BACI designed experiment we investigated the impact of sublethal pesticide exposure on oxidative stress biomarkers protein carbonyl and DNA damage (8-OHdG), in an arid-zone lizard species, *Pogona vitticeps*. A single ecologically relevant dose of pesticide was applied via oral gavage to treatment animals. Lizard condition, activity measures, and blood biomarkers were measured at relevant sampling intervals. Cholinesterase (ChE) and acetylcholinesterase (AChE) enzymatic biomarkers were measured in response to fenitrothion, and fipronil blood residues were measured for fipronil-treated lizards. Results suggested no significant treatment effect of either pesticide on parameters measured, however, 8-OHdG levels decreased by ≥ 45% for both pesticide treatment groups and not controls. Protein carbonyl levels showed a high degree of individual variation that proved more influential than pesticide exposure. Building our understanding of the macromolecular impacts of sublethal pesticide exposure on wild lizard populations is an integral step in addressing the current gap in literature and management practices. Our study has also highlighted the complex nature of studying oxidative stress in the field and the sheer necessity of future study.

## Introduction

Oxidative stress is integral in key biological processes across all taxa (Costantini 2019). Investigating and understanding oxidative stress in living organisms is complex and multi-faceted. It is well understood that the oxidative stress system is based on a careful balance between eustress and damage, the status of this balance determining the level of oxidative stress present at any given time (Finkel and Holbrook 2000). Reactive oxygen species (ROS) are in a continuous cycle over the life of an organism and are kept under threshold through endogenous and exogenous antioxidant defence systems (Sies 1997, Sies et al. 2017). Once this balance is broken there is a potential for detrimental oxidative stress which targets various areas of the body. Variation in individual personality and physiology, disease status, reproductive status, age, sex, external environment and climate, xenobiotics and anthropogenic changes all influence oxidative stress levels and must be accounted for (Isaksson et al. 2011, Beaulieu and Costantini 2014). Furthermore, as oxidative status is reflective of life history traits and the environment an organism inhabits, we can use tailored biomarkers to inform us about the status of organisms in that environment. Oxidative stress biomarkers have the potential to be extremely useful in identifying the impact that anthropogenic threats, such as xenobiotic compounds, have on a population. This information can be used to formulate relevant guidelines for the conservation of threatened species (Beaulieu and Costantini 2014).

Pesticides are a key anthropogenic stressor that affect populations of wild animals worldwide (Gibbons et al. 2000, Todd et al. 2010, Fasola et al. 2021). They are used in many aspects of human life, from pest control in the home to large scale spray operations to protect agricultural assets (Simon-Delso et al. 2015). Both fipronil (a phenyl-pyrazole insecticide) and fenitrothion (an organophosphate pesticide) are commonly and widely used pesticides globally: fipronil in particular contributes significantly to the world insecticide market (Simon-Delso et al. 2015). Fipronil (5-amino-3-cyano-1-(2,6-dichloro-4-trifluoromethylphenyl)-4-trifluoromethylsulfinyl pyrazole, CAS 120068-37-3) targets critical processes in the central nervous system (CNS) and disrupts the action of gamma-amino butyric acid (GABA). This is done via binding to post-synaptic receptors that block essential chloride channels necessary for GABA innervation (Hainzl et al. 1998, Narahashi 2010, Story et al. 2021). Fenitrothion (O,O-dimethyl-O-(3-methyl-4-nitrophenol)-phosphorothioate, CAS 122-14-5) similarly targets the CNS, but also the peripheral nervous system (PNS). Specifically, fenitrothion targets cholinergic innervation through the inhibition of cholinesterase (ChE) activity most importantly impacting acetylcholinesterase (AChE) activity (Story and Cox 2001, Weir et al. 2010). Both pesticides are expected to cause oxidative stress (Banerjee et al. 2001, Gardner and Oberdorster 2016, Shah and Parveen 2022), by inactivation or down-regulation of antioxidant defence systems decreasing the antioxidant potential of cells or increasing ROS levels through biotransformation to electrophilic or free-radical intermediates (Banerjee et al. 2001, Lushchak 2011). It is possible to detect these macromolecular changes through the use of oxidative stress biomarkers, which have the ability to detect early-stage impacts to environmental contaminants, such as pesticides (Mitchelmore et al. 2005).

Proteins, lipids and DNA are the key targets for oxidative stress. These macromolecule systems are important markers of oxidative stress and are used widely in oxidative stress research (Kaur et al. 2014). Although lipid peroxidation is widespread, it is highly susceptible to free radical attack of any kind (Butler et al. 2016). Eating increases circulating triglyceride levels in the blood which can be reflected in higher levels of lipid peroxidation (Pérez-Rodríguez et al. 2015) which is termed postprandial oxidative stress (Sies et al. 2005, Wallace et al. 2010). Given unknown eating habits of wild organisms, lipid peroxidation is not a particularly useful measure for measuring oxidative stress when sampling wild populations. In contrast, protein carbonyl has been suggested as a good proxy for generalised oxidative stress (Reznick et al. 1992, Shah and Parveen 2022). As proteins are highly abundant and present at high concentration in cells, they are a major targets and likely sites for oxidative damage (Davies 2005, Davies 2016, Hawkins and Davies 2019). Reactive oxygen species target proteins rapidly resulting in early formation of carbonyls throughout the oxidative stress timeline. Biochemical modifications cause post-translational changes that alter amino acids causing changes in protein structure and function (Dalle-Donne et al. 2003, Gianazza et al. 2007). Potential oxidative damage is skewed toward protein modification in many situations, and thus is a more specific marker for measuring oxidative stress and damage (Davies 2005, Davies 2016). DNA damage caused by oxidative stress is also a widely used and reliable biomarker of interest due to its links to genetic mechanisms related to aging, as well as many biological disorders (Nikitaki et al. 2015). Damage to DNA involves the formation of DNA crosslinks via oxidation, methylation, depurination and deamination and basic structural changes as a result of single and double-stranded DNA breaks (Sies 1997, Kaur et al. 2014). It is widely accepted that the most important DNA lesions and thus biomarkers are those associated with hydroxylation and modification of purine and pyrimidine bases (Slaninova et al. 2009). This is generated as ROS attacks DNA at the C-8 position of 2-deoxyguanosine in turn forming the DNA lesion 8-hydroxy-2-deoxyguanosine or 8-OHdG (Kaur et al. 2014). Furthermore, 8-OHdG impacts mechanisms used for the regulation of gene expression by altering the methylation of cytosines (via enzyme-catalysation) (Thompson 2004, Valko et al. 2007). As it is the most critical DNA lesion across taxa with high prevalence, 8-OHdG is an ideal biomarker of oxidative stress to assess individual and population changes in response to sublethal pesticide exposure (Thompson 2004, Slaninova et al. 2009). Protein carbonyl and 8-OHdG are ideal markers to select and build on previous studies on reptiles (Amaral et al. 2012, Mingo et al. 2017, Isaksson 2020, Fasola et al. 2021).

Oxidative stress research is generally lacking across reptilian taxa. With global declines in reptile species, it is important to explore whether xenobiotics or environmental contamination, particularly pesticides, are a key factor (Gibbons et al. 2000, Todd et al. 2010). Reptiles, specifically squamates are susceptible to both indirect and direct impacts of pesticide exposure given their life history traits and ecosystem role as secondary consumers (Sparling et al. 2010, Gardner and Oberdorster 2016, Contador-Kelsall et al. 2022). Lizards are excellent bioindicators of environmental pollution and reveal valuable information about the ecosystems they inhabit (Campbell and Campbell 2002, Silva et al. 2020). The handful of studies investigating the impact of pesticides on oxidative stress levels in reptiles have only begun to illuminate the complexity of this topic. The wide selection of biomarkers available in conjunction with the use of different sample types and timing creates a vast number of questions that need to be answered. To address some of these questions, we have conducted a field-based experiment to investigate the impact of sublethal pesticide exposure on a range of physiological, behavioural, and molecular biomarkers of oxidative stress in a widespread, arid-zone, native Australian lizard, the Central Bearded Dragon (*Pogona vitticeps*, Ahl 1926). Biomarkers of oxidative stress have been selected across all levels of biological organisation and sampled along a time series to build a comprehensive picture of such effects. We expect that sublethal pesticide exposure will alter oxidative stress status, but we will also build baseline information and validate the use of protein carbonyl and 8-OHdG as appropriate biomarkers of oxidative stress for field-based research.

## Materials and methods

### Study species and site

*Pogona vitticeps* is a widespread, medium-bodied, terrestrial arid-zone agamid species that inhabits semi-arid and arid Australia (Cogger 2018). *Pogona vitticeps* are semi-arboreal omnivores that inhabit various desert habitats, dry sclerophyll forests, cypress pine woodlands, *Acacia* scrubs, and eucalypt woodlands (Rej and Joyner 2018). This species occurs in rangelands potentially subject to pesticide applications used in locust control (Bain et al. 2004).

The study was conducted from mid-January until mid-March 2019 at Nombinnie Nature Reserve (33°04’25.34”S, 145°47’28.39”E). Nombinnie Nature Reserve is situated in central-western NSW in Australia and consists of 70,000 ha of Mallee, White Cypress Pine, Bimble and Red Box, and Belah woodlands amongst lignum and other vegetation (NSW National Parks and Wildlife Services 2022). Mean monthly temperatures from January to March in 2019 were 32.5, 25.6, and 22.7 °C respectively, with the highest temperature recorded at 46.9 °C in January 2019. Historically (1885–2009) mean spring–summer (September–March) rainfall was 224.7 mm (Australian Bureau of Meteorology 2021). In spring – summer 2017/2018 total rainfall for the area was 165.5 mm and in 2018/2019 total rainfall was 220.4 mm (Australian Bureau of Meteorology 2021).

### Animal capture, handling, and tracking

*Pogona vitticeps* were captured, sampled, and released between 17 days – 1 day pre-exposure to pesticide, as described in Contador-Kelsall et al. (2022). Lizards were initially captured opportunistically during visual scanning surveys. Once captured, lizards were morphologically sexed by hemipenal inspection, and snout to vent length (SVL) and mass (g) were measured to determine scaled body mass indices (SBMI) (Peig and Green 2009). A blood sample of 200 µL was taken from the caudal tail vein. Whole blood was partitioned for haemoglobin (Hb) measurement, blood spot cards (PerkinElmer 226 Five Spot cards) and haematocrit tubes for later plasma collection. Further methodology detailing processing of blood samples can be found in the Appendix. Following sampling, a Sirtrack VHF tag (Sirtrack Ltd, Havelock North, New Zealand) and HOBO accelerometer (HOBO® Pendant G, Onset Computer Corporation) were fixed together and attached to each lizard’s tail dorsally using brown surgical tape. Pilot data suggested lizards were rarely active during the night (Bernich et al. 2022) so accelerometers were set to record data points every 30 s during daylight.

Following initial capture, lizards were sampled at planned sampling sessions for the duration of the experiment. Sampling sessions consisted of; post 24 h, post 7 days, post 14 days, and post 28 days after pesticide dosing. For all subsequent sampling we located each lizard using their allocated VHF tag and took body measurements (SVL, mass (g)) and a blood sample (200 μL). At the final sample session (post 28 d) VHF tags and accelerometers were removed permanently.

### Pesticide dosing

To create an ecologically relevant, sublethal pesticide dose, dose was calculated based on known pesticide residue levels found on natural vegetation and insect prey (after aerial spraying) with maximum feeding rates of *P. vitticeps*, and the average dietary intake of an adult lizard (30% insect, 70% vegetation) from unpublished feeding data and previous pesticide deposition literature (Szabo 2005, Story et al. 2013). In field studies, mean fenitrothion residue levels were found to be 62.0 µg/g on vegetation and 39.8 µg/g on locusts (Story et al. 2013) and fipronil residue levels were between 1.4 and 5.6 µg/g on vegetation (1.4 µg/g used in this experiment) and 0.124 µg/g on locusts (Szabo 2005, Story et al. 2021). According to treatment, lizards received 16.60 mg/kg of fenitrothion or 0.51 mg/kg of fipronil in a single dose. A stock solution was formulated for each treatment based on this dosage rate, with exact individual doses calculated after the initial sampling session once body mass (g) was recorded.

Lizards were allocated to one of 3 treatments at random, with fipronil (*n* = 5), fenitrothion (*n* = 5), and technical grade corn oil (control, *n* = 6). Both pesticides were suspended in technical grade corn oil, to mimic the formulations used in aerial spraying (Kitulagodage et al. 2011). Fipronil was first dissolved in < 150 µL acetone given its low water solubility (acetone solubility = 54.6 g/100 mL) (Tingle et al. 2003) and then suspended in technical grade corn oil. A Covidien Kendall™ plastic feeding tube (6 x 410 mm) was used to deliver pesticides and/or corn oil via oral gavage.

### Biomarker assays

#### Fipronil

All samples were prepared according to Raju et al. (2016) and previously described in Contador-Kelsall et al. (2022). An Agilent 6490 Triple Quad Liquid Chromatography – mass spectrometry (LCMS) was used to analyse all samples. Fipronil parent and metabolite compounds (fipronil, fipronil desulfinyl, fipronil sulfide, and fipronil sulfone) standards were purchased from Sigma-Aldrich. The R^2^ for each calibration curve was above 0.996. All samples were run in negative mode and clearly defined, confirmed by their most abundant product ions at optimised collision energies. To ensure there was no contamination throughout, a positive control (0.01 μg/mL fipronil) and a negative control (acetonitrile) were run every five samples.

#### Fenitrothion

All plasma samples were analysed using the Ellman assay (Ellman et al. 1961), as modified by Gard and Hooper (1993), and described in detail in Buttemer et al. (2008) and Contador-Kelsall et al. (2022). An optimum dilution ratio of 1:5 was applied to all samples. Samples were run in duplicates or triplicates dependent of sample availability. Blank samples without the addition of enzyme and mouse serum (Sigma-Aldrich, Australia) were used as a between-assay standard. Total plasma cholinesterase (TChE) and acetylcholinesterase (AChE) activity were directly measured from the plasma samples. Butrylcholinesterase (BChE) activity was then calculated as the difference between TChE and AChE. Exposure to fenitrothion was indicated as a suppression in TChE and AChE when compared to control samples.

### Quantifying protein carbonyl

Prior to protein carbonyl analysis, we performed a bicinchoninic acid assay (BCA) to quantify protein in each frozen plasma sample. Quantification was performed using Pierce™ Rapid Gold BCA Protein Assay Kit (ThermoFisher Scientific, Sydney, Australia) following the microplate procedure using the FLUOstar Omega plate reader (BMG Labtech, Ortenberg, Germany). Each sample was run as a duplicate and absorbance was read at 450 nm, with the assay range limits between 20 to 2000 µg/mL. Each plasma sample was diluted according to BCA assay results at 10 µg/mL in 1X PBS in preparation for the protein carbonyl assay. Once diluted the samples were used within 24 h to prevent freeze-thaw cycle degradation of samples.

Oxidative stress was measured via protein carbonyl derivatives using the OxiSelect™ Protein Carbonyl ELISA kit (Cell biolabs, San Diego, CA). The ELISA was conducted according to the manufacturer’s protocol, with the final incubation time of 4–6 min, prior to adding the stop solution. Each sample was run in triplicate and an average used for final analysis. Absorbance was read at 450 nm using the FLUOstar Omega plate reader. Omega MARS data analysis software (BMG Labtech, Ortenberg, Germany) performed best-fit analysis and created a standard curve based on protocol standards, including standard 8 as the blank, where 3^rd^ Polynomial fit based on blank corrected data was selected according to the equation: *Y* = *offset* + *c*1*x* + *c*2*x*^2^ + *c*3*x*^3^.

### Quantifying DNA Damage (8-Hydroxy-2’-deoxyguanosine)

Frozen plasma samples were used for the DNA Damage Competitive ELISA kit (ThermoFisher Scientific, Sydney, Australia). Prior to running the ELISA, we created a dilution series of plasma with 1X Assay Buffer ranging from 1:4 to 1:64, based on (Olsson et al. 2012) . All plasma samples were diluted to optimal dilution (1:16) with 1X Assay Buffer prior to the assay. All samples, standards and blanks were run in triplicate for the ELISA, with controls run in duplicate. Standards 1–9 were diluted as per manufacturer’s instructions, in a serial dilution ranging from 8 000 pg/mL to 0 pg/mL. The ELISA was run according to the manufacturer’s protocol, with absorbance read at 450 nm 10 min after adding the Stop Solution using the FLUOstar Omega plate reader. Omega MARS data analysis software was used for best-fit analysis based on a 4-parameter best fit and created a standard curve based on protocol standards. Standard curve was created according to the equation:

$$Y = \frac{{Bottom + \left( {Top - Bottom} \right)}}{{\left( {1 + \left( {\frac{{EC50}}{x}} \right)^{slope}} \right)}}$$

### Activity measures

Activity datapoints were measured every 30 s in g-force on an X and Y axis. To ensure no confounding impacts of human disturbance, we removed data surrounding capture and release. All data points from 10 min before capture and 20 min after release were removed from the dataset. We categorised a threshold for movement according to the smallest amount of variance that represented lizard movement, 0.0199 g^2^ based on Bernich et al. (2022) and assessed data accordingly. Remaining data for each sample session was grouped in 10-minute intervals and then added into daily totals of minutes moved from 6:00–19:00. Three consecutive 24 h periods were selected either before or after (depending on sample session) all set sampling times to create the dataset for activity and ensure consistency across each sample session. According to each activity dataset, a mean of total minutes moved for each session was recorded and used as the final activity measurement.

### Statistical analysis

To determine which variables influence protein carbonyl and DNA damage (8-OHdG) levels in *P. vitticeps*, we measured 6 different explanatory variables. These variables were: treatment (control, fipronil, fenitrothion), time, mean daily temperature, scaled body mass index (SBMI), haemoglobin (Hb), and activity. A total of 13 lizards were used to analyse the protein carbonyl dataset (*n* = 4–5 per treatment) and 14 lizards used to analyse the DNA damage dataset (*n* = 4–6 per treatment). Protein carbonyl analysis was based on three sample time points: Pre, Post 24 h, Post 7 d, of which four individuals had a time point missing (total dataset = 35). DNA damage analysis was based on four sample time points: Pre, Post 7 d, Post 14 d, and Post 28 d, where four individuals had 1–2 missing time points (total dataset = 49).

We ran a Pearson correlation matrix for all continuous explanatory variables in both protein carbonyl and DNA damage datasets to assess collinearity between variables. Most explanatory variables assessed for DNA damage models had r-values < 0.5, based on the understanding that Pearson correlation coefficient > 0.5 indicated a highly correlated set of variables (Zuur et al. 2009). Time and temperature were significantly positively correlated (*R* = 0.50, *P* < 0.05) and therefore temperature was removed from the model prior to analysis. In the protein carbonyl dataset, explanatory variables time and temperature were also significantly negatively correlated (*R* = −0.68, *P* < 0.001) and thus temperature was removed as an explanatory variable prior to further analysis. Mauchly’s test of sphericity was performed for the DNA damage dataset and no assumptions were violated (*P* > 0.05).

Model selection via backward elimination was used to select the best-fit model to explain variation in both protein carbonyl and DNA damage levels. Linear mixed-effect models for repeated measures were used to analyse the datasets. The lme4 (Bates et al. 2015) and lmerTest (Kuznetsova et al. 2017) packages were used in R Studio (ver 1.4.1717, RStudio Team 2021). The MuMin package (Bartoń 2022) was used to obtain marginal and conditional R^2^ and the AICcmodavg package (Mazerolle 2020) was used to obtain AICc values. All models were run using REML and Satterthwaite’s method of approximation. Full candidate models included all explanatory variables, treatment, time, and their interaction term, activity, SBMI, and Hb as fixed effects, with lizard ID as a random effect. Candidate full models were as follows:

Protein carbonyl: lmer(Protein Carbonyl ~ Treatment*Time + Activity + SBMI + Hb + *(1|Lizard ID), data* = *PC)*

DNA damage: lmer(DNA Damage ~ Treatment*Time + Activity + SBMI + Hb + *(1|Lizard ID), data* = *DNA)*

Using guidance from AICc (Akaike’s information criterion values corrected for a small sample size) and R^2^ values each explanatory variable was removed until significance was found, if applicable. Multiple best-fit models were selected, including those with lowest AICc score (Tables 1, 3).

**Table 1:** Selected best-fit models explaining influence of explanatory variables (model terms) ranging from treatment, time, scaled body mass index (SBMI), activity, and haemoglobin (Hb) on protein carbonyl levels in *P. vitticeps* (*n* = 11, total observations = 20)

Model	Model terms	Numerator df	Denominator df	F	P	*R*^*2*^*m*	*R*^*2*^*c*	*AIC*_*c*_
a) PC ~ Treatment*Time + SBMI + Hb + Activity + (1|Lizard ID)	Treatment	2	6.624	0.125	0.885	19.7%	86.7%	88.99
	Time	1	7.268	0.103	0.758			
	Treatment*Time	2	5.730	0.791	0.497			
	SBMI	1	8.611	1.721	0.224			
	Activity	1	5.835	0.354	0.574			
	Hb	1	10.990	1.369	0.267			
b) PC ~ SBMI + Hb + Activity + (1|Lizard ID)	Activity	1	7.619	1.414	0.270	19.1%	85.0%	46.36
	SBMI	1	13.163	3.099	0.102			
	Hb	1	15.878	1.163	0.297			

*df* Degrees of freedom, *F* F statistic, *P* P-value, *R*^*2*^*m* Marginal R^2^, *R*^*2*^*c* Conditional R^2^ and *AICc* Hurvich and Tsai’s Criterion are a result of a restricted maximum likelihood model, where individuals are repeatedly sampled over time

To investigate the influence cholinesterases (ChE) and acetylcholinesterase (AChE) (fenitrothion-treated group) and fipronil sulfone (fipronil-treated group) on protein carbonyl and DNA damage levels we similarly ran linear mixed-effect models. Each model was run independently with either fipronil sulfone, ChE, or AChE as explanatory variables. Models with ChE and AChE also included a treatment interaction term, as these datasets were inclusive of control lizards (Tables 2, 4).

**Table 2:** Linear mixed-effect models investigating the relationship between biomarkers of exposure including fipronil sulfone (*n* = 4, total observations = 7), ChE (*n* = 7, total observations = 13), AChE (*n* = 7, total observations = 12) and protein carbonyl levels in *P. vitticeps*

Model	Model terms	Numerator df	Denominator df	F	*P*	*R*^*2*^*m*	*R*^*2*^*c*
a) PC ~ Fipronil sulfone + (1|Lizard ID)	Fipronil Sulfone	1	2.056	1.662	0.323	2.6%	9.3%
b) PC ~ Treatment * ChE + (1|Lizard ID)	Treatment	1	4.993	0.000	0.995	2.4%	75.7%
	ChE	1	4.950	0.139	0.725		
	Treatment*ChE	1	4.950	0.001	0.973		
c) PC ~ Treatment*AChE + (1|Lizard ID)	Treatment	1	7.970	0.653	0.442	8.9%	70.5%
	AChE	1	7.910	0.001	0.983		
	Treatment*AChE	1	7.910	0.739	0.415		

*df* Degrees of freedom, *F* F statistic, *P* P-value, *R*^*2*^*m* Marginal R^2^, *R*^*2*^*c* Conditional R^2^ are a result of a restricted maximum likelihood model, where individuals are repeatedly sampled over time

## Results

### Protein Carbonyl

Individual physiological measures, SBMI and Hb differed regardless of treatment and time for lizards used in the protein carbonyl analysis (*n* = 11). SBMI ranged from 241.5 g to 422.6 g with a mean (±SD) of 320 g ± 42.8 g and Hb ranged from 69 g/L to 102 g/L with a mean (± SD) of 89.1 g/L ± 9.1 g/L. Change in protein carbonyl varied from −41–68% with a mean (±SD) of 7 ± 30%. Activity measures ranged from 53.3 to 246.7 mins across all individuals and all sample sessions. The mean (± SD) activity was 142.1 min ± 56.7 min.

**Fig. 1:**
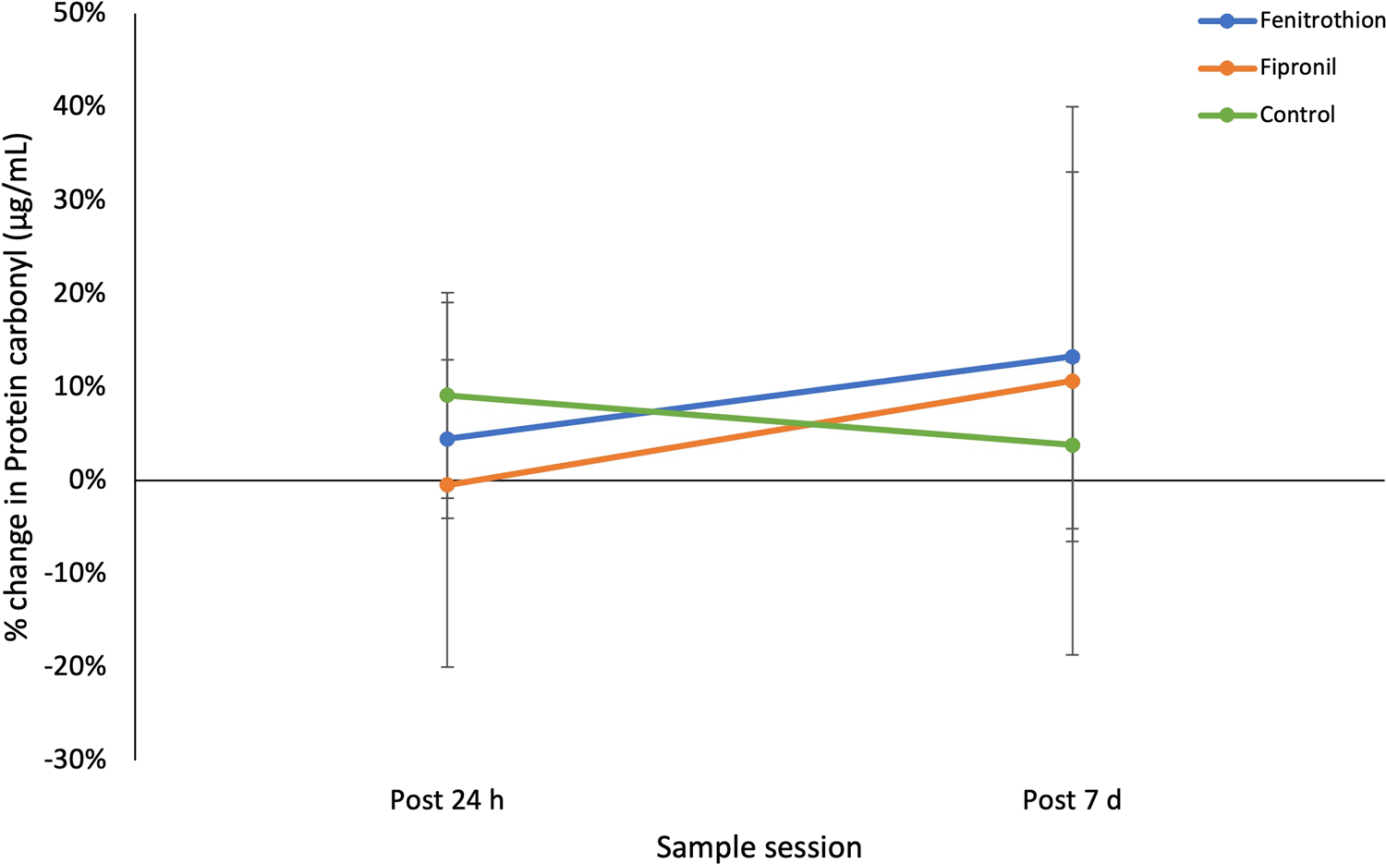
Means plot (± SE) depicting the % change in protein carbonyl (μg/mL) levels over time for each treatment group in *Pogona vitticeps*. Fenitrothion-treated lizards shown in blue (*n* = 4), firpronil-treated lizards shown in orange (*n* = 4), and control lizards shown in green (*n* = 5)

Protein carbonyl levels (% change from pre-dose levels) did not vary significantly among treatments and across time (Table 1a). Despite backward elimination all resulting models failed to explain changes in protein carbonyl levels. Individual variation was large, as evidenced by the standard errors (Fig. 1). Furthermore, SBMI, activity and Hb. did not significantly influence protein carbonyl levels in *P. vitticeps*. The full model had a marginal R^2^ of 19.7% and a conditional R^2^ of 86.7% (Table 1a), suggesting individual variation across subjects is responsible for protein carbonyl level fluctuations, more so than any explanatory variable. Even in the reduced model (SBMI, Hb, and activity as explanatory variables, Table 1b) there were comparable R^2^ values confirming that individual variation across subjects explains a large portion of the model. Protein carbonyl levels in fenitrothion-treated lizards increased from 4% at post 24 h to 13% at post 7 d. Similarly, fipronil-treated lizards increased from 0% at post 24 h to 11% at post 7 d. Control lizards saw a decrease from 9% at Post 24 h to 4% at post 7 d.

**Fig. 2:**
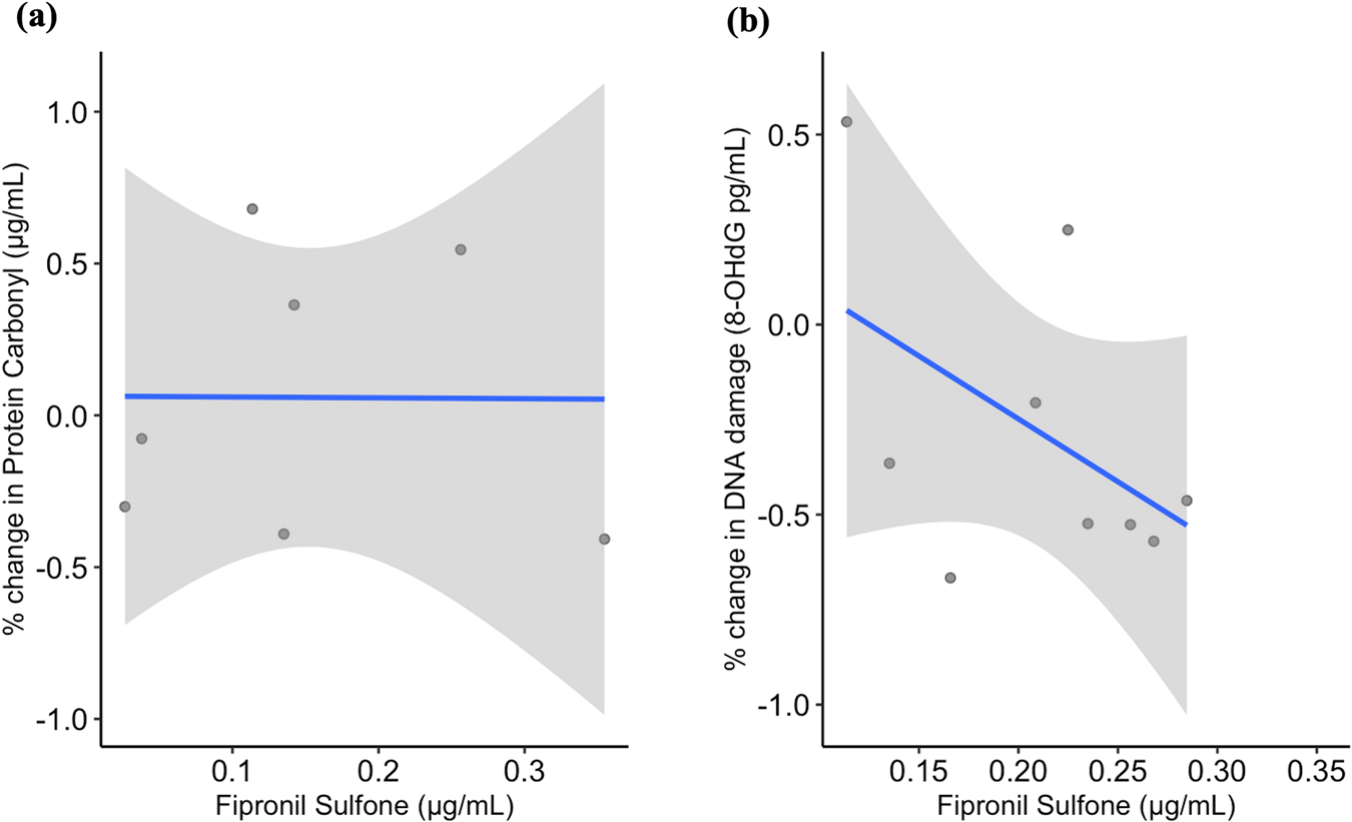
The relationship between fipronil sulfone (μg/mL) levels and (**a**) % change in protein carbonyl (μg/mL) or (**b**) % change in DNA damage (8-OHdG pg/mL) levels in *Pogona vitticeps* (*n* = 4) aside from treatment. ± SE is shown in grey shading and linear trend line in blue

Fipronil sulfone (metabolite of fipronil) was present in all treatment lizard blood samples and absent in all control lizards for both Post 24 h and Post 7 d. Mean values (± SD) for fipronil-treated lizards were 0.069 μg/mL ± 0.06 for Post 24 h and 0.2149 μg/mL ± 0.097 for Post 7 d. Fipronil sulfone levels increased by 212% from Post 24 h to Post 7 d. No significant relationship was detected between fipronil sulfone residue levels and protein carbonyl levels (Table 2a, Fig. 2a). Marginal R^2^ and conditional R^2^ were both very low (Table 2a).

**Fig. 3:**
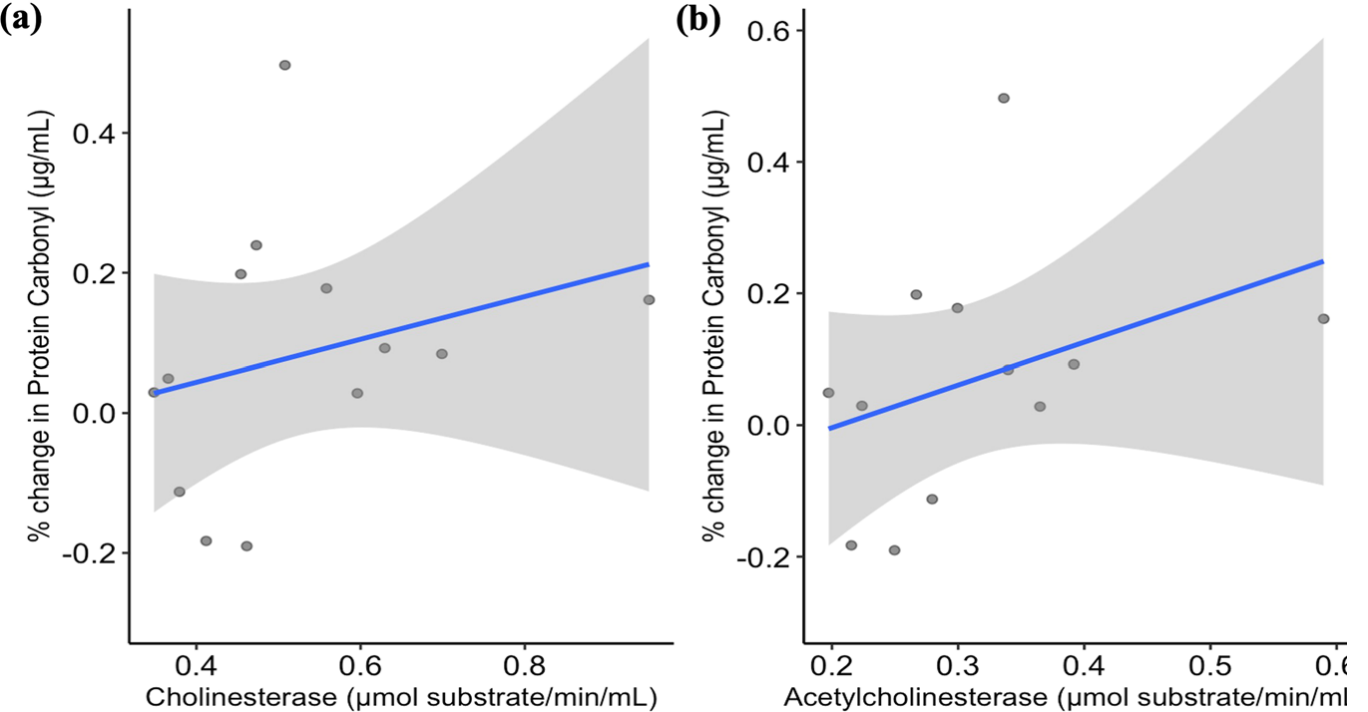
Relationship between % change in protein carbonyl (μg/mL) levels and (**a**) cholinesterase (μmol substrate/min/mL) or (**b**) acetylcholinesterase (μmol substrate/min/mL) in *Pogona vitticeps* (*n* = 7) aside from treatment. Linear trend line shown in blue and ± SE shown by the grey shading

ChE and AChE levels varied similarly in both fenitrothion treated and control groups. Fenitrothion-treated lizards saw a 12% decrease in ChE activity levels from Post 24 h to Post 7 d and a 13% decrease for AChE activity levels. Control lizard ChE activity levels decreased by 3% from Post 24 h to Post 7 d and AChE activity levels increased by 3%. As ChE and AChE was decreasing for fenitrothion-treated lizards, protein carbonyl levels were increasing by 9% over time, conversely to a decrease in protein carbonyl levels of 5% for control lizards. However, neither ChE nor AChE influenced protein carbonyl levels in *P. vitticeps* (Table 2b, c, Fig. 3). A marginal R^2^ and conditional R^2^ values for the ChE and protein carbonyl model were very similar to the AChE model suggesting large levels of individual variation.

### DNA damage (8-OHdG)

Physiological parameters (SBMI and Hb) varied among individuals across treatments used in the DNA damage analysis (n = 14). SBMI ranged from 241.5 g to 463.3 g with a mean (± SD) of 328.9 g ± 43.5 g. Hb ranged from 69 g/L to 112 g/L ± 9.7 g/L, with a mean (±SD) of 87.4 g/L. DNA damage (8-OHdG) ranged from a −80 to 160% change from initial sample (Pre-dose), with a mean (± SD) of 54 ± 30% change. Activity measures ranged widely from 0 min (mins) – 206.6 min across all time periods with an overall mean (± SD) of 105.2 min ± 53.6 min.

**Fig. 4:**
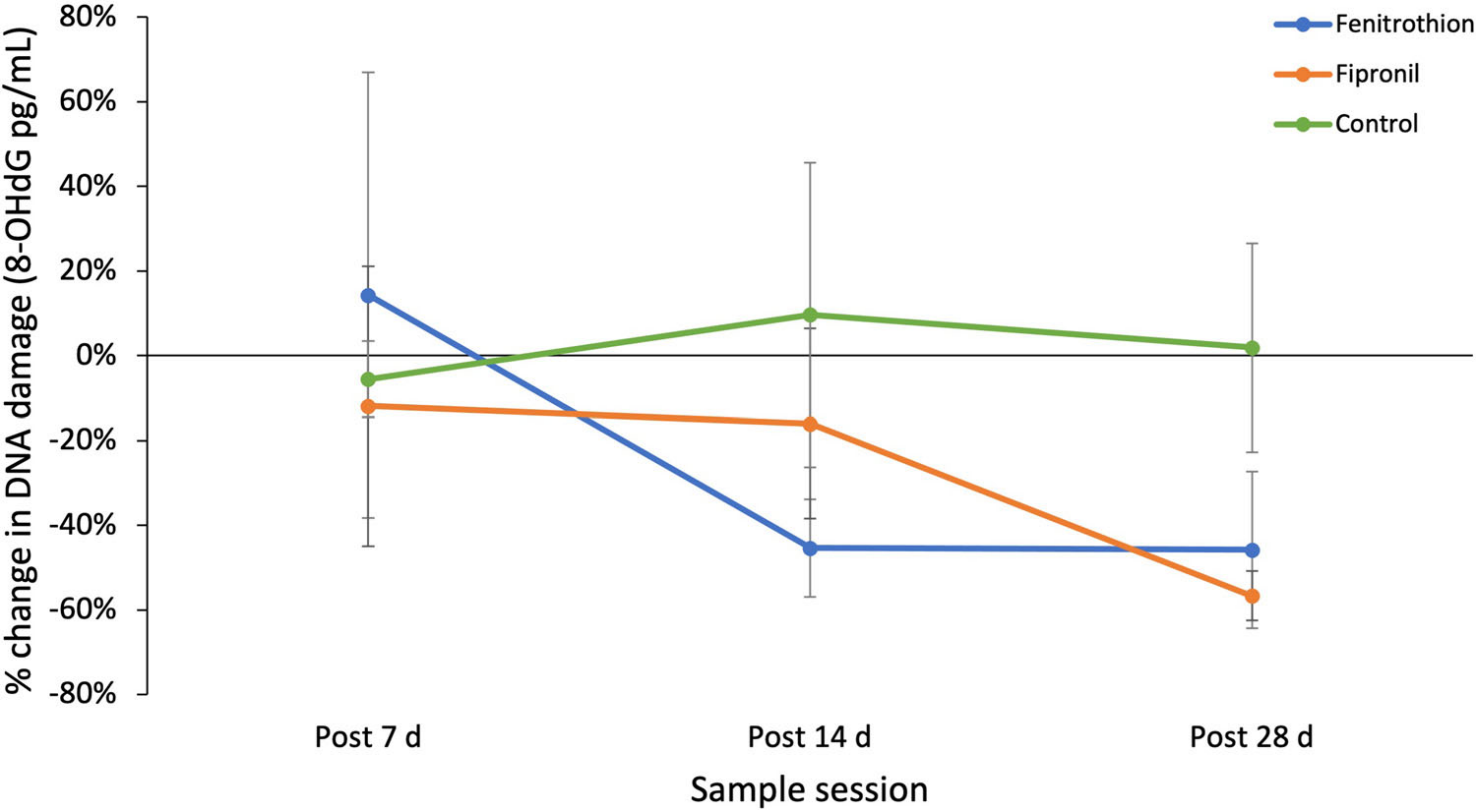
Means plot (± SE) depicting the % change in DNA damage (8-OHdG pg/mL) levels over time for each treatment group in *Pogona vitticeps*. Fenitrothion-treated lizards shown in blue (*n* = 4), fipronil-treated lizards shown in orange (*n* = 4), and control lizards shown in green (*n* = 6)

**Fig. 5:**
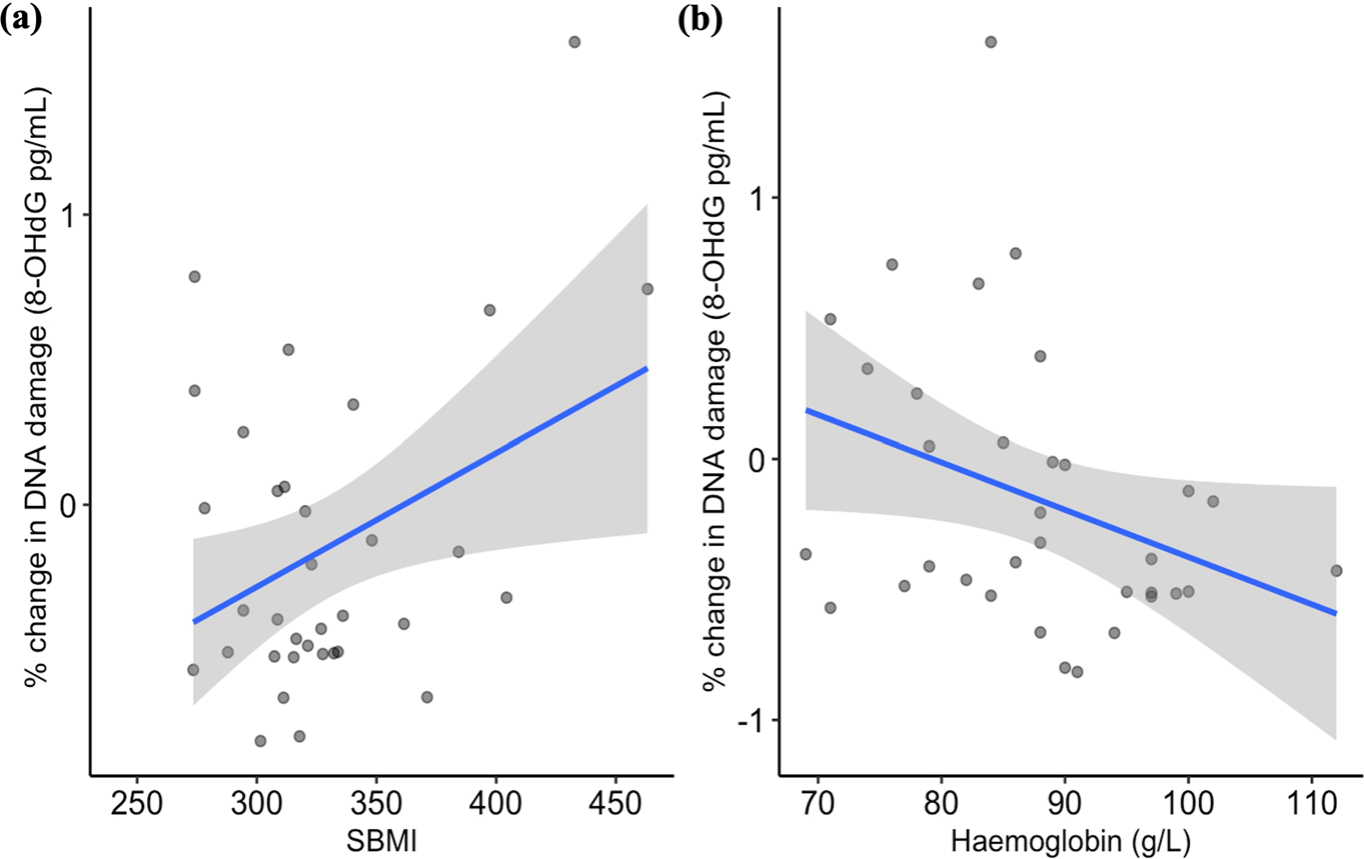
The relationship between DNA damage (8-OHdG pg/mL) and (**a**) Scaled body mass index (SBMI (g)) or (**b**) Haemoglobin (g/L) over 28–42 days in *Pogona vitticeps* (*n* = 40, *n* = 36 respectively). Standard error is shown in grey shading, and linear trend line in blue

While there was no significant effect of treatment or time on 8-OHdG levels in *P. vitticeps* (Table 3), DNA damage levels (% change from pre-dose levels) decreased across time for both fenitrothion and fipronil-treated lizards (Fig. 4). From post 7 d to post 28 d 8-OHdG decreased 60% for lizards in the fenitrothion treatment group. Fipronil-treated individuals showed a 45% reduction in 8-OHdG levels from post 7d to post 28 d. Control lizards fluctuated from an initial increase at post 14 d by 16% followed by a decrease of 8% at post 28 d. The full model prior to backward elimination resulted in a marginal R^2^ of 38.5% and a conditional R^2^ of 47.7% (Table 3a). SBMI significantly increased 8-OHdG levels in models including treatment, activity, Hb and subject as explanatory variables (See Appendix, also Table 3b). The best model to explain DNA damage levels in *P.vitticeps* included only SBMI and Hb (Table 3c). Both SBMI and Hb significantly influence 8-OHdG levels in *P. vitticeps* independent of treatment effect or time (P < 0.05, Fig. 5). There was a significant positive relationship between SBMI and 8-OHdG levels and a significant negative relationship between Hb and 8-OHdG levels (Table 3c).

**Table 3:** Selected best-fit models explaining influence of explanatory variables (model terms) ranging from treatment, time, scaled body mass index (SBMI), activity, and haemoglobin (Hb) on DNA damage (8-OHdG) levels in *P. vitticeps* (*n* = 14, total observations = 34)

Model	Model terms	Numerator df	Denominator df	F	*P*	*R*^*2*^*m*	*R*^*2*^*c*	*AIC*_*c*_
a) DNAD ~ Treatment*Time + SBMI + Hb + Activity + (1|Lizard ID)	Treatment	2	8.269	1.361	0.308	38.5%	47.4%	120.68
	Time	2	15.321	0.676	0.523			
	Treatment*Time	4	15.168	0.571	0.688			
	SBMI	1	8.158	4.606	0.064			
	Activity	1	18.638	0.601	0.448			
	Hb	1	11.835	2.986	0.986			
b) DNAD ~ Treatment + SBMI + Hb + (1|Lizard ID)	Treatment	2	8.775	1.668	0.243	34.4%	44.2%	81.01
	SBMI	1	8.930	5.888	0.038*			
	Hb	1	14.952	3.520	0.080			
c) DNAD ~ SBMI + Hb + (1|Lizard ID)	SBMI	1	12.009	6.067	0.029*	28.5%	43.8%	75.86
	Hb	1	19.875	4.641	0.044*			

Significant effects are printed in bold*. *df* Degrees of freedom, *F* F statistic, *P* P-value, *R*^*2*^*m* Marginal R^2^, *R*^*2*^*c* Conditional R^2^ and *AICc* Hurvich and Tsai’s Criterion are a result of a restricted maximum likelihood model, where individuals are repeatedly sampled over time

Fipronil sulfone was measured in control and fipronil-treated lizards. Fipronil sulfone was present in all treatment lizards for all sample sessions, including post 28 d at the end of the experiment, and was absent from all control lizards for the duration of the experiment. Mean values (± SD) for treatment individuals include, 0.215 μg/mL ± 0.11 (Post 7 d), 0.223 μg/mL ± 0.01 (Post 14 d), and 0.239 μg/mL ± 0.06 (Post 28 d). On average sulfone levels remained high at similar levels for all time periods, with an increase of 11% at post 28 d from post 7 d. There was no significant relationship between fipronil sulfone levels and DNA damage in fipronil-treated *P. vitticeps* (*P* > 0.05, Table 4a, Fig. 2b).

**Table 4:** Linear mixed-effect models investigating the relationship between biomarkers of exposure including fipronil sulfone (*n* = 4, total observations = 9), ChE (*n* = 9, total observations = 21), AChE (n = 10, total observations = 24) and DNA damage (8-OHdG) levels in *P. vitticeps*

Model	Model terms	Numerator df	Denominator df	F	*P*	*R*^*2*^*m*	*R*^*2*^*c*
a) DNAD ~ Fipronil sulfone + (1|Lizard ID)	Fipronil Sulfone	1	7.000	2.145	0.186	21.1%	21.1%
b) DNAD ~ Treatment * ChE + (1|Lizard ID)	Treatment	1	17.000	0.112	0.743	22.7%	22.7%
	ChE	1	17.000	4.537	0.048*		
	Treatment*ChE	1	17.000	0.033	0.859		
c) DNAD ~ Treatment*AChE + (1|Lizard ID)	Treatment	1	17.656	0.002	0.966	9.7%	24.5%
	AChE	1	18.419	0.001	0.973		
	Treatment*AChE	1	18.419	0.117	0.737		

Significant effects are printed in bold*. *df* Degrees of freedom, *F* F statistic, *P* P-value, *R*^*2*^*m* Marginal R^2^, and *R*^*2*^*c* conditional R^2^ are a result of a restricted maximum likelihood model, where individuals are repeatedly sampled over time

**Fig. 6:**
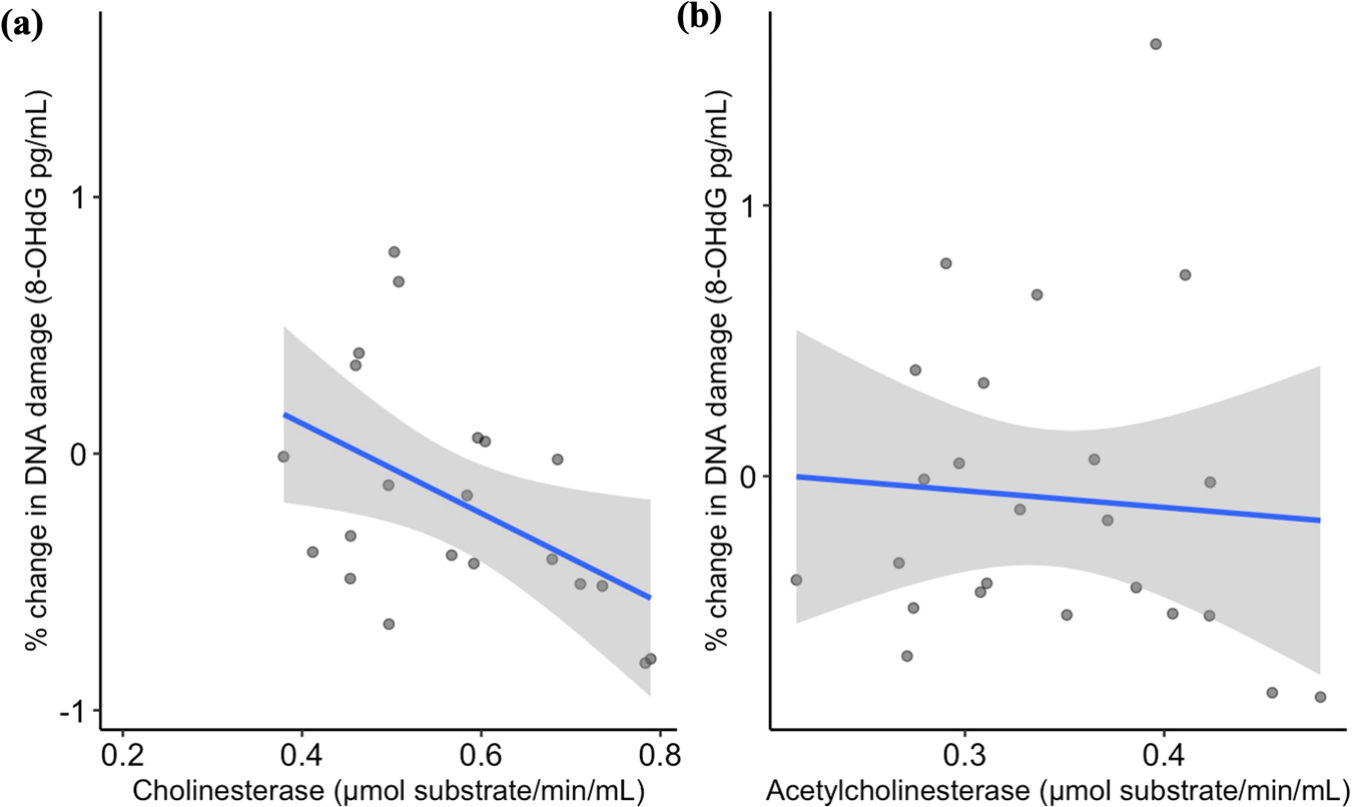
Relationship between % change in DNA damage (8-OHdG pg/mL) levels and (**a**) cholinesterase (μmol substrate/min/mL) or (**b**) acetylcholinesterase (μmol substrate/min/mL) in *Pogona vitticeps* (*n* = 10) including individuals from fenitrothion and control groups. Linear tend line shown in blue and ± SE shown by the grey shading

Both ChE and AChE activity levels were measured in control and fenitrothion-treated lizards. ChE levels were significantly negatively correlated with 8-OHdG levels (*P* < 0.05, Table 4b, Fig. 6), where treatment and ChE interaction term did not (*P* > 0.05, Table 4b). There was no significant effect of AChE or its interaction term with treatment in explaining 8-OHdG levels (Fig. 6, Table 4c). In the fenitrothion treatment group ChE increased 7% between Post 7 d and Post 14 d and decreased slightly to a 5% change at Post 28 d. AChE activity levels increased by 16% from Post 7 d to Post 14 d, and further increased at Post 28 d at 36%. Control lizards on the other hand showed an increase of 13% in ChE between Post 7 d and Post 14 d, and a 15% increase at Post 28 d. Control lizard AChE activity levels plateaued at a 17% increase from Post 7 d to both Post 14 d and Post 28 d.

## Discussion

Our results investigating the degree of oxidative stress in pesticide-treated lizards were not greater than natural background levels for both macromolecular (protein carbonyl and 8-OHdG) and enzymatic biomarkers (AChE). Individual variation accounted for a higher level of oxidative damage than sublethal exposure to pesticides, however, non-significant trends in DNA damage (8-OHdG) levels provide some evidence of a relationship with pesticide exposure. As the study was based on wild *P. vitticeps* individuals we were unable to account for potential drivers of these high levels of individual variation, such as biological and reproductive age, microclimate, disease, and food availability and it is integral to keep this in mind when interpreting the results (Monaghan et al. 2009, Kaur et al. 2014). There is also the potential for a compounding effect of multiple stressors resulting in the oxidative stress levels portrayed in the current study (Bertram et al. 2022). However, utilising a repeated measures time series sampling protocol in our experiment provides some accountability in investigating oxidative stress levels throughout the duration of the experiment (Fasola et al. 2021, Ritchie and Friesen 2022). Our results are therefore cautiously encouraging; ecologically relevant exposure to pesticides may not result in evidence of severe oxidative damage in *P. vitticeps*.

Despite no significant treatment effect on either of the macromolecular biomarkers (PC and 8-OHdG), there is evidence of a trend in relation to 8-OHdG levels. Both pesticide treatment groups show a ≥45% decrease in 8-OHdG levels from post 7 d to post 28 d, whereas control treatment group does not substantially change; evidence of pesticide exposure generating oxidative stress (Fig. 4). For example, fipronil-treated individuals show a plateau in 8-OHdG levels from post 7 d to post 14 d, followed by a further drop in 8-OhdG levels from post 14 d to post 28 d which correlates with peak fipronil sulfone levels in the blood of treated individuals. The secondary decrease in 8-OHdG levels after post 14 d in the fipronil-treated cohort could be the result increasing pesticide metabolites, as fipronil sulfone is more detrimental and toxic to body systems than fipronil itself (Hainzl et al. 1998, Peveling and Demba 2003). Any decrease seen in both treatment groups primarily suggests the mobilisation of the antioxidant defence system in response to oxidative stress (Sies 1997, Sies et al. 2017, Severo et al. 2020). As fipronil sulfone reaches its peak, it is probable there is an increase in oxidative stress, and thus we see an upregulation of the antioxidant defence system. This likely causes a decrease in 8-OHdG levels as a result of enzymatic antioxidant defences, such as superoxide dismutase (Winterbourn 2016), preventing any oxidative damage to DNA. In contrast, fenitrothion has no secondary metabolites that are more toxic (Shore and Douben 1994), so we see a faster decrease in 8-OHdG levels of 45% at Post 14 d indicating potentially a quicker mobilisation of antioxidant defence systems. The decrease in 8-OHdG levels and assumed upregulation of endogenous antioxidants can be described as eustress and may be an explanation for the results seen for fipronil and fenitrothion-exposed individuals (Sies et al. 2017, Hawkins and Davies 2019), however the potential link between decreased levels and fenitrothion exposure is less clear. The overarching enzyme group, cholinesterase’s, or B esterases indicate a negative relationship between ChE activity levels and 8-OHdG levels. There is no obvious relationship between 8-OHdG and AChE, the primary target of OPs (Thompson 1999), which suggests that this interaction is simply between pre-existing ChE activity and 8-OHdG levels, which we would expect to be present regardless of OP exposure. It remains unclear why ChE significantly correlates to 8-OHdG but we have shown there is no significant influence of sublethal fenitrothion exposure on these levels, despite a trend indicating the upregulation of antioxidant defence systems. Future investigation into the relationship between these markers, both aside from OP exposure and when subjected to higher doses of fenitrothion would yield useful information.

There are multiple pathways that pesticides, such a fipronil or fenitrothion can take to cause oxidative stress. Reactive oxygen species (ROS) created via excessive cycling of cytochrome P450 enzymes, decreased antioxidant potential or downregulation of cellular antioxidants, and alterations to the function of mitochondria (decreasing ability to metabolise and detoxify) all result in oxidative stress to lipids, proteins, and DNA (Banerjee et al. 2001, Lushchak 2011). Additionally, these xenobiotic compounds can join redox cycles and transforming cells so that they become electrophilic (Banerjee et al. 2001, Lukaszewicz-Hussain 2010, Lushchak 2011). However, it is unlikely exposure to fipronil or fenitrothion resulted in a decrease in antioxidant potential given the suggested upregulation evident in changes in 8-OHdG levels in both treatment groups (Fig. 4). Thus, as it is implied that some oxidative stress (namely 8-OHdG) has occurred in *P. vitticeps* due to pesticide exposure, we suggest fipronil and fenitrothion potentially disrupt cytochrome P450 enzyme systems and/or interfere in key cellular processes within mitochondria or processes associated with transcription or translation (Kitulagodage et al. 2011, Lushchak 2011, Kaur et al. 2014, Isaksson 2015). Measuring these parameters would be very interesting to further understand how ecologically relevant pesticides may impact oxidative stress levels in lizards. Laboratory methodology has been developed to assess changes in mitochondrial function and cytochrome P450/NADPH-cytochrome P450 reductase (Guengerich et al. 2009, Stier et al. 2017, Kowaltowski 2019). Measuring cytochrome P450 enzymes is relatively easy however it must be done in the laboratory with specialised equipment but is a good candidate for future research in this field (Guengerich et al. 2009). On the other hand, mitochondrial function and oxidants are more difficult to measure, and technology is still developing (Kowaltowski 2019). Stier et al. (2017) developed a method to accurately measure mitochondrial function using red blood cells, which could be applied to field studies with reptiles in the future.

In the laboratory and field, we know pesticides cause oxidative stress, and we would expect an increase as a result of exposure (Valdivia et al. 2007, Lushchak 2011, Amaral et al. 2012, Severo et al. 2020). This was evident in 8-OHdG levels in treatment lizards, yet not so for PC levels. Aside from high levels of individual variation dictating PC levels in our experiment we should also consider that the sampling protocol used in our study to detect PC levels (Pre, Post 24 h, Post 7 d) was suboptimal for detecting any potential significant changes. Significant fluctuations in PC levels can occur and return to baseline in as short as 24 h or as long as 7 days depending on the intensity of the stressor, and these temporary impacts can be easily missed (Nikolaidis et al. 2012, Costantini 2019, Isaksson 2020, Marins et al. 2021). While our sampling protocol measured the predicted critical periods, as suggested by the literature, any key changes occurring before post 24 h and between post 48 h – post 7 d may have been missed. Sample type specific to oxidative stress biomarkers is also critical for measuring correct levels of oxidative stress in response to external stressors. For example, Wu et al. (2015) showed that Zebra fish (*Danio rerio*) had increased levels of ROS in gills, brain and liver tissue in response to cold temperatures, however the time ROS peaked was different depending on the tissue type. Similarly, South-American Catfish (*Rhamdia quelen*) exposed to a mixture of pesticides at environmental concentrations saw an increase in protein carbonyl levels in liver and brain tissues but a decrease in gills and muscle (Marins et al. 2021). It is likely that in our study, alternate sample types may have shown higher levels of oxidative stress. In terms of sample type, blood is still utilised widely and accepted as a good generalised marker indicative of oxidative status throughout the body (Margaritelis et al. 2015). Margaritelis et al. (2015) concluded that majority of oxidative stress biomarkers measured in blood adequately reflected oxidative status in tissues. This was true for; reduced glutathione, SOD, CAT, Malondialdehyde, glutathione peroxidase, and vitamin C and E but not so for oxidised glutathione and reduced/oxidised glutathione ratio. Argüelles et al. (2004) found that protein carbonyl levels in blood correlated with heart and liver tissue, but not spleen and kidney, indicating once again the variable nature of oxidative stress. Additionally, as blood is highly integrated throughout the body, it has the potential to have the most variable response to oxidative stress (Isaksson 2015, Speakman et al. 2015, Ouyang et al. 2018, Costantini 2019). At present, using blood to measure oxidative stress changes exhibits methodology that is relatively non-invasive and most importantly non-lethal. Any alternate analysis of various tissues (for example, liver and spleen) would require the subject to be euthanised, which was not the intention in this study. Using blood samples also allow the analysis of a time series, which is incredibly informative when investigating oxidative stress.

This study is one of only a handful to investigate a collection of biomarkers of oxidative stress in a remote field setting (Amaral et al. 2012, Mingo et al. 2017, Fasola et al. 2021, Simbula et al. 2021) and the first to investigate ecologically relevant impacts of pesticides on an important Australian reptile. There is a necessity to continue growing this data because measuring oxidative stress status is incredibly complex. While we found indications of an upregulation of antioxidant defences, evidenced namely in 8-OHdG results, we were unable to use antioxidant (non-enzymatic or enzymatic) biomarkers in our study as these require quick access to laboratory conditions, a situation not available in the field (Selman et al. 2012). The stability of these biomarkers decreases over time depending on storage protocols in conjunction with intrinsic reactivity (Jansen et al. 2013, Pérez-Rodríguez et al. 2015). Additionally, the levels portrayed in these assays may not in fact reflect accurate antioxidant responses based on the complex interactions existing among antioxidants (Pérez-Rodríguez et al. 2015), a further reason for not including such assays in our study. Instead, we selected feasible biomarkers that would not degrade in the field and chose times relevant to oxidative stress time points.

Individual physiological traits of scaled body mass index (SBMI) and haemoglobin (Hb) significantly influenced DNA damage (8-OHdG) levels. Our results suggest that as body condition increases so does DNA damage (Fig. 5a). Page and Stuart (2012) demonstrated that body mass was the key variable that explained variation in DNA repair mechanisms in endotherms. Oligonucleotide ligation activities and nucleotide incorporation was found to be negatively correlated with body mass. Our results show a positive relationship between the two variables, the converse of Page and Stuart (2012) however we see similarity in the significant correlations between body condition and oxidative stress in DNA. A candidate hypothesis as to why SBMI and DNA damage may be related is age. Age is a key factor that must be investigated when examining body condition and oxidative stress levels (Hoekstra et al. 2020). There is conflicting evidence for ectotherms as to whether age is correlated with oxidative stress levels, as they do not consistently fit into the oxidative stress theory of ageing (Buttemer et al. 2010). Hoekstra et al. (2020) summarises all available studies that present correlations or absence of between macromolecular damage and age in reptiles. Specifically for DNA damage, a handful of studies have found a positive correlation with age (Bronikowski 2008, Robert and Bronikowski 2010, Schwartz and Bronikowski 2013), while some have shown no association with age (Woodhead et al. 1980, Regan et al. 1982, Schwartz and Bronikowski 2011). For example, Robert and Bronikowski (2010) found that long-lived ecotypes of Western Terrestrial Garter Snakes (*Thamnophis elegans*) were in fact smaller than the short-lived ecotypes, and experienced higher DNA damage, but the damage was repaired more efficiently. Additionally, the smaller, long-lived ecotypes had more efficient antioxidant defence systems and mitochondria. Evident here, and in our study, is a complex relationship between body size, condition, age, and oxidative stress, damage, and repair, where a multitude of future studies are needed to help clarify such relationships. It has been demonstrated that reptiles in particular pose more complexity in understanding the correlations between physiology and oxidative stress given the nature of their life histories. External environment, in particular temperature influences the physiology and behaviour of reptiles and in turn metabolic rate and growth, adding a level of intricacy to understanding SBMI and oxidative stress levels (Buttemer et al. 2010).

**Fig. 7:**
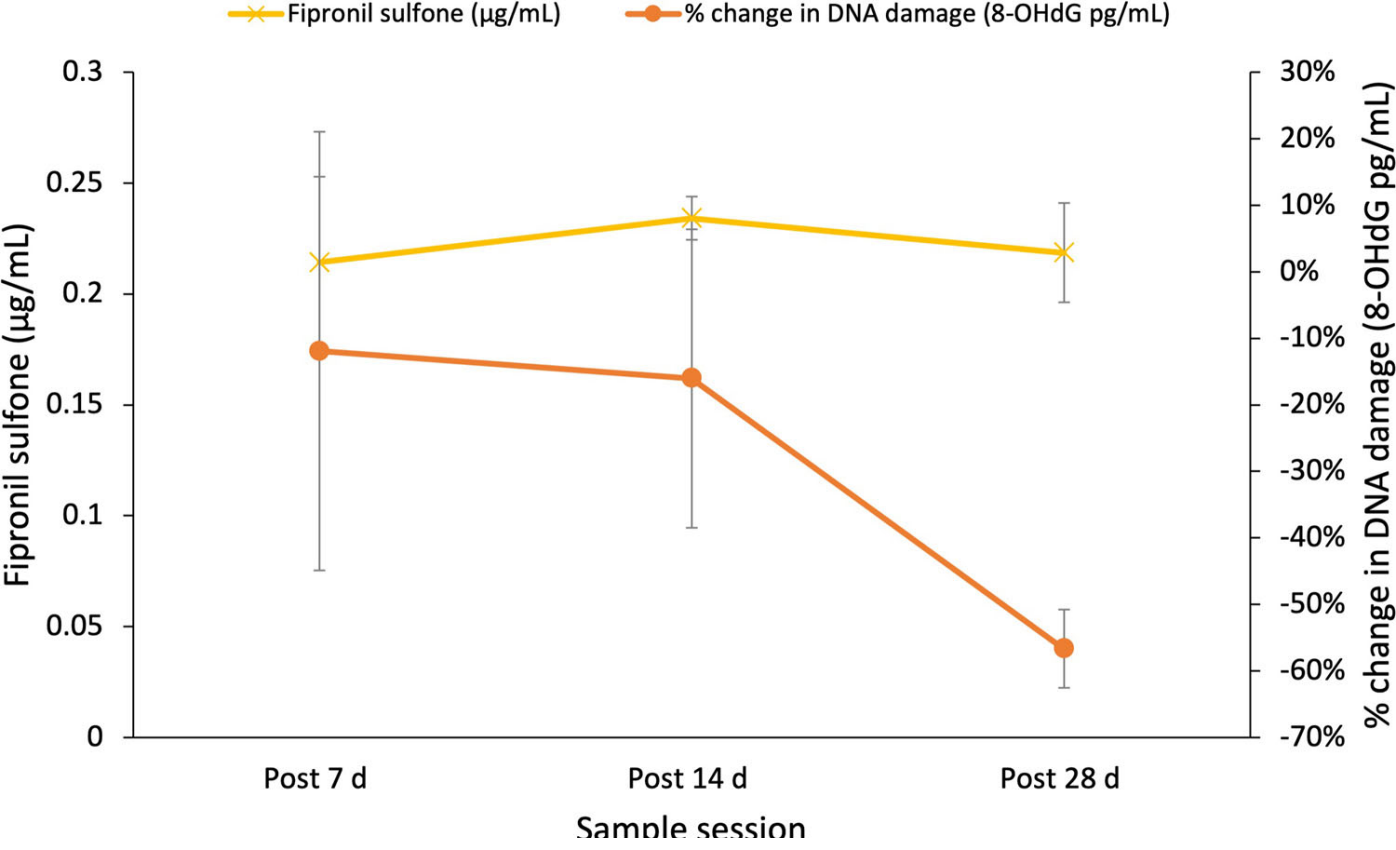
Mean fipronil sulfone (μg/mL) and % change in DNA damage (8-OHdG pg/mL) in *Pogona vitticeps* (*n* = 4) over time. Error bars ± SE

**Fig. 8:**
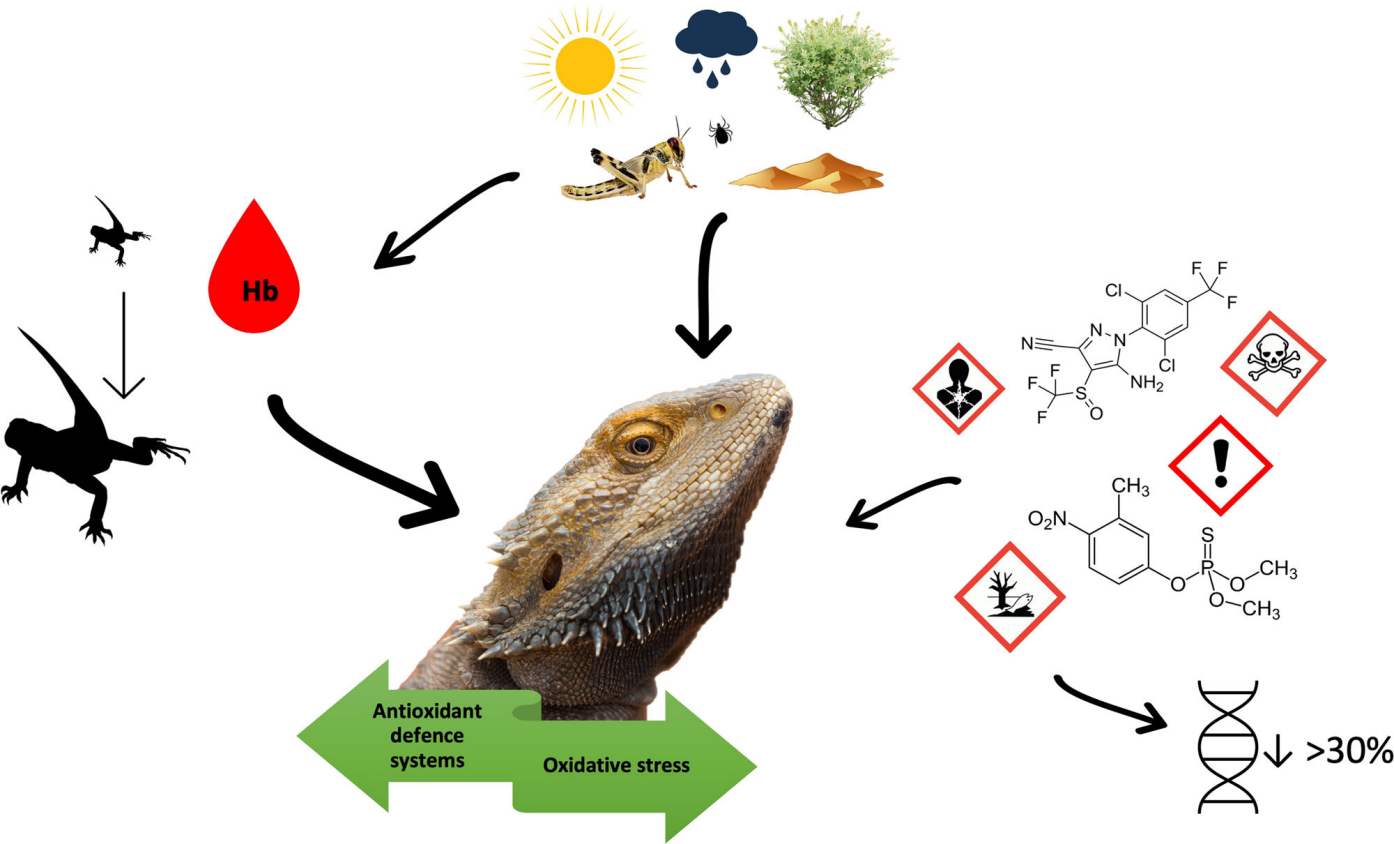
Infographic diagram showing the balancing act between antioxidant defence systems and oxidative stress levels influenced in our study by individual variation (e.g., SBMI and Hb) linked to environmental parameters (diet, climate, disease), and sublethal pesticide exposure. Arrows indicate strength of interactions

As haemoglobin levels (Hb) increased 8-OHdG levels decreased (Fig. 5b). Hb can be used in part to describe the fitness and health of a reptile, tending to increase with better fitness (Zayas et al. 2011, Minias 2015, Johnstone et al. 2017). Oxidative stress causes damage to red blood cells (RBC) and the denaturation of haemoglobin molecules (Mohanty et al. 2014). We hypothesise that the healthier the lizard the lower the levels of oxidative stress. Bauerová et al. (2017) and Herrera-Dueñas et al. (2014) both found that oxidative stress was detected in RBCs, in conjunction with lower Hb and packed cell volume (PCV) concentrations in birds that inhabited urban areas opposed to rural areas. This is consistent with Goodchild et al. (2022) where Song Sparrows (*Melospiza melodia*) inhabiting suburban areas displayed lower Hb concentrations compared to individuals inhabiting rural areas. However, the study did not find any significant differences in any of the oxidative stress markers tested (GSH, d-ROMs, HOCI neutralisation) between sites, which could suggest Hb concentrations are not dictated directly by urbanisation. Cid et al. (2018) also detected significant decreases in Hb concentrations in House Sparrows (*Passer domesticus*) that were exposed to sublethal doses of lead (Pb). It is probable that our results are also demonstrating a degree of impact on Hb concentrations because of a sublethal pesticide dose, or potentially alternate stressors, given there is no obvious treatment effect (Figs. 7, 8).

We can equate the level of oxidative stress witnessed in our study to that described by Lushchak (2014), as basal oxidative stress (BOS) witnessed in our protein carbonyl results and low intensity oxidative stress (LOS) witnessed in our DNA damage results. Based on this proposed classification system for oxidative stress (based on intensity), BOS represents levels that have no observable impacts caused by the applied stressor and cannot be detected by current methods, a description suited to our protein carbonyl findings. On the other hand, DNA damage clearly showed molecular changes associated with probable increases in antioxidant defence systems (Keap1/nrf2 system upregulation) but with no endpoint parameter changes (increases in 8-OHdG), placing the level of oxidative stress experienced between BOS and LOS (Lushchak 2014). It is evident that a sublethal pesticide dose has likely caused a small increase in oxidative stress, resulting in the upregulation of endogenous antioxidant defence systems. Additionally, there is strong evidence that individual physiological factors and life history traits influence oxidative stress levels, potentially to a larger extent, in wild *P. vitticeps*. Wild individuals and populations are inherently difficult to study due to the many external factors that cannot be accounted for (Beaulieu and Costantini 2014, Kaur et al. 2014). Despite this, there is urgent need for field-based, ecologically relevant studies so that a relevant understanding of basic oxidative stress responses to not only anthropogenic stressors, such as sublethal pesticide exposure, but also the extent external environment and individual biology influences these levels. Oxidative stress influences critical biological functioning related to all aspects of life and can predict the survivability and reproductive capacity of a species (Isaksson et al. 2011, Beaulieu and Costantini 2014). It is imperative to understand this relationship when assessing impacts on wild reptile populations. The lack of ecologically relevant field studies is impeding formulation of guidelines that can be used for reptile conservation (Ritchie and Friesen 2022). Our study has successfully investigated various biomarkers of oxidative stress ranging from cellular level to whole body physiological changes in response to sublethal exposure to pesticides. Importantly, the study has validated protein carbonyl and 8-OHdG as biomarkers of oxidative stress in Central Bearded Dragons (*P. vitticeps*) in a wild setting. We have built upon similar previous studies, further investigating potential informative markers, and most significantly demonstrating that the time at which a sample is taken is a crucial element of biomarker selection that must be included in all future studies.

## Supplementary information

Supplementary Appendix
