# Supplementary material for: Individual variation within wild populations of an arid-zone lizard dictates oxidative stress levels despite exposure to sublethal pesticides
Source: Ecotoxicology. 2023 Apr 26;32(4):470–86. doi: 10.1007/s10646-023-02653-8 (PMC10199878; doi:10.1007/s10646-023-02653-8)
Supplement: Supplementary file 1 — Supplementary Appendix [file 10646_2023_2653_MOESM1_ESM.docx]

**Individual variation within wild populations of an arid-zone lizard dictates oxidative stress levels despite exposure to sublethal pesticides.**

Isabella Contador-Kelsall, Kimberly Maute, Maxwell De Beer, Kristine French

**Additional methodology**

**Blood processing**

Haemoglobin was measured immediately after the blood sample was taken using a Hemocue HB 201 (Abgleholm, Sweden). Haematocrit tubes containing whole blood were processed on the day of collection to collect plasma. Haematocrit tubes were centrifuged at 14 000 RCF for 2.5 minutes (Hematospin 1300, Hawksley and Sons Ltd) and plasma collected via sterile needle and syringe. All blood samples were frozen at -20°C whilst in the field due to logistics and transferred at the end of the study to -80°C.

**Additional information regarding protein carbonyl protocol**

We used 3rd polynomial fit based on blank corrected data, with equation

$$Y=offset+c1x+c2x^{2}+c3x^{3}$$

Where for plate 1 run on 11/9/2019:

offset = 0.019514

c1 = 0.289731

c2 = -0.02066

c3 = -0.00033

r = 0.995256

$\boldsymbol{r}^{\boldsymbol{2}}$ ***= 0.990534***

Plate 2 run on 12/12/2019:

offset = -0.00164

c1 = 0.197361

c2 = 0.0038

c3 = -0.00126

r = 0.999555

$\boldsymbol{r}^{\boldsymbol{2}}$ ***= 0.99911***

**Additional information regarding DNA damage (8-OHdG) protocol**

We used 4-parameter fit based on blank corrected data, with equation

$$Y=\frac{Bottom+(Top-Bottom)}{(1+\left( \frac{EC50}{x} \right)^{Slope})}$$

Where for plate 1 run on 16/12/2020:

Top = 1.383521

Slope = - 1.10085

EC50 = 882.5504

Log(EC50) = 2.94574

Bottom = 0.049166

r = 0.99948

$\boldsymbol{r}^{\boldsymbol{2}}$ ***= 0.998961***

Plate 2 run on 21/12/2020:

Top = 1.432192

Slope = - 1.08586

EC50 = 841.383

Log(EC50) = 2.924994

Bottom = 0.029708

r = 0.999667

$\boldsymbol{r}^{\boldsymbol{2}}$ ***= 0.999334***

**Table A1.** Akaike’s information criterion model rankings for different candidate models investigating the influence of scaled body mass index (SBMI), activity, haemoglobin (Hb), time and treatment on protein carbonyl levels in *P. vitticeps*.

| Model Terms | AIC_c_ | ∆AIC_c_ | Wt | Cum.Wt |
| --- | --- | --- | --- | --- |
| Intercept only | 10.53 | 0.00 | 1 | 1 |
| PC ~ SBMI + (1\|Lizard ID) | 23.19 | 12.66 | 0 | 1 |
| PC ~ Activity + SBMI + (1\|Lizard ID) | 35.33 | 24.80 | 0 | 1 |
| PC ~ Activity + SBMI + Hb + (1\|Lizard ID) | 46.36 | 35.83 | 0 | 1 |
| PC ~ Time + Activity + SBMI + Hb + (1\|Lizard ID) | 53.15 | 42.62 | 0 | 1 |
| PC ~ Treatment + Time + Activity + SBMI + Hb + (1\|Lizard ID) | 67.88 | 57.35 | 0 | 1 |
| PC ~ Treatment*Time + Activity + SBMI + Hb + (1\|Lizard ID) | 88.99 | 78.46 | 0 | 1 |

∆AIC_c_: the difference in AIC_c_ compared with the model with the lowest AIC_c_, Wt: weight of the model according to ∆AIC_c_, Cum Wt: cumulative model weights.

**Table A2.** Akaike’s information criterion model rankings for different candidate models investigating the influence of scaled body mass index (SBMI), activity, haemoglobin (Hb), time and treatment on DNA-damage (8-OHdG) levels in *P. vitticeps*.

| Model Terms | AIC_c_ | ∆AIC_c_ | Wt | Cum.Wt |
| --- | --- | --- | --- | --- |
| Intercept only | 62.16 | 0.00 | 1 | 1 |
| DNAD ~ SBMI + Hb + (1\|Lizard ID) | 75.86 | 13.70 | 0 | 1 |
| DNAD ~ Treatment + SBMI + Hb + (1\|Lizard ID) | 81.01 | 18.85 | 0 | 1 |
| DNAD ~ Treatment + Activity + SBMI + Hb + (1\|Lizard ID) | 92.32 | 30.16 | 0 | 1 |
| DNAD ~ Treatment + Time + Activity + SBMI + Hb + (1\|Lizard ID) | 102.23 | 40.07 | 0 | 1 |
| DNAD ~ Treatment*Time + SBMI + Hb + (1\|Lizard ID) | 105.57 | 43.41 | 0 | 1 |
| DNAD ~ Treatment*Time + Activity + SBMI + Hb + (1\|Lizard ID) | 120.68 | 58.52 | 0 | 1 |

Models in *bold* include significance for SBMI. ∆AIC_c_: the difference in AIC_c_ compared with the model with the lowest AIC_c_, Wt: weight of the model according to ∆AIC_c_, Cum Wt: cumulative model weights.

**Table A3.** A selected best-fit model to investigate the effect treatment, scaled body mass index (SBMI), haemoglobin (Hb) and activity on DNA-damage (8-OHdG) levels in *P. vitticeps* (n = 14, total obs = 34).

| Model | Model terms | Numerator df | Denominator df | F | P | *R^2^m* | *R^2^c* | *AIC_c_* |
| --- | --- | --- | --- | --- | --- | --- | --- | --- |
| DNAD ~ Treatment + SBMI + Hb + Activity + (1\|Lizard ID) | Treatment | 2 | 8.068 | 1.769 | 0.231 | 37.6% | 49.4% | 92.32 |
|  | Activity | 1 | 25.318 | 1.231 | 0.278 |  |  |  |
|  | SBMI | 1 | 8.690 | 5.856 | **0.040*** |  |  |  |
|  | Hb | 1 | 14.070 | 2.855 | 0.113 |  |  |  |

Notes: Significant effects are printed in bold*. Degrees of freedom (df), F statistic (F), P-value (P), marginal R^2^ (R^2^m), conditional R^2^ (R^2^c) and Hurvich and Tsai’s Criterion (AICc) are a result of a restricted maximum likelihood model, where individuals are repeatedly sampled over time.
